# Allogeneic hematopoietic stem cell transplantation in two brothers with DNA ligase IV deficiency: a case report and review of the literature

**DOI:** 10.1186/s12887-019-1724-z

**Published:** 2019-10-11

**Authors:** Sarah Schober, Karin Schilbach, Michaela Doering, Karin M. Cabanillas Stanchi, Ursula Holzer, Patrick Kasteleiner, Jens Schittenhelm, Juergen F. Schaefer, Ingo Mueller, Peter Lang, Rupert Handgretinger

**Affiliations:** 1 Department I – General Pediatrics, Hematology/Oncology, University Children’s Hospital Tuebingen, Hoppe-Seyler-Str.1, 72076 Tuebingen, Germany; 2 Department of Neuropathology, Institute of Pathology and Neuropathology, Eberhard-Karls University Tuebingen, Calwer Str. 3, 72074 Tuebingen, Germany; 3 Department of Diagnostic and Interventional Radiology, University Hospital Tuebingen, Hoppe-Seyler-Str. 3, 72076 Tuebingen, Germany; 4 Division for Pediatric Stem Cell Transplantation and Immunology, Clinic for Pediatric Hematology and Oncology, University Medical Center Hamburg-Eppendorf, Martinistr, 52, 20246 Hamburg, Germany

**Keywords:** DNA ligase IV deficiency, LIG4, Severe combined immunodeficiency, SCID, Steroid-refractory graft-versus-host disease, GvHD, Conditioning regimen, Hematopoietic stem cell transplantation, HSCT, Cyclosporine A

## Abstract

**Background:**

DNA ligase IV deficiency is a rare autosomal recessive disorder caused by hypomorphic mutations in the DNA ligase IV (LIG4) gene. DNA ligase IV is an essential protein for the development of a healthy immune system as well as for the protection of genomic integrity. Apart from typical stigmata, patients with DNA ligase IV deficiency are characterized by progressive bone marrow failure and a predisposition to malignancy. To our knowledge this reported case is the first description of two brothers with ligase IV deficiency who are treated with different hematopoietic stem cell transplantation (HSCT) regimens resulting in vastly divergent outcomes.

**Case presentation:**

The cases of two brothers suffering from severe recurrent infections and growth retardation are described. The laboratory findings showed pancytopenia with significant lymphopenia. The two boys were diagnosed with DNA ligase IV deficiency, associated with severe combined immunodeficiency (SCID). Both patients received HSCT from two different matched unrelated donors (MUD) at the age of 33 and 18 months. The older brother succumbed post-transplant due to fatal side-effects 143 days after allogeneic HSCT. The younger brother – conditioned with a different regimen – received a T cell depleted graft 4 months later. No severe side-effects occurred, neither post-transplant nor in the following years. Ten years after HSCT the patient is well off, living a normal life and attending a regular high school. His immune system is fully reconstituted, resulting in a maximum of T cell receptor (TCR) diversity, which is a prerequisite for immune competence. However, he still suffers from microcephaly, dwarfism and dystrophy.

**Conclusions:**

This case report gives an example of a successful HSCT as a treatment option in a genetic disorder such as ligase IV deficiency, using a rather mild conditioning regimen. Further studies are required to determine the viability and efficacy of this treatment option.

## Background

DNA ligase IV deficiency is a rare form of autosomal recessive, radiosensitive severe combined immunodeficiency (SCID), which is caused by hypomorphic mutations of the DNA ligase IV (LIG4) gene. The disorder was first described in 1999 [1]. Less than 45 cases have been reported worldwide [2–8]. Considering the number of published descriptions of asymptomatic patients with DNA ligase IV mutations, there might be an indefinite number of unreported cases [5]. The clinical manifestation varies from asymptomatic mutation carrier status to severe immunodeficiency with life-threatening infections and lethal malignancies [3, 5, 8].

Typical clinical features are microcephaly, growth retardation beginning in utero, typical facial appearance (“bird-like face” with sloping forehead, micrognathia, long nose), developmental and mental delay [9], combined immunodeficiency, pancytopenia due to bone marrow failure (70% of all cases reported), clinical and cellular hypersensitivity to ionizing radiation and a predisposition to malignancy (25% of all cases reported, especially leukemia and lymphoma) [3, 8, 10]. Bony deformations and skin conditions have also been reported [11]. These symptoms show high clinical variability.

DNA ligase IV is a key component in the repair of DNA double-strand breaks by the non-homologous end-joining (NHEJ) pathway. This pathway is also required for generation of T and B cell receptors and for processing the diversity of specific immunoglobulins [10, 11]. Thus, DNA ligase IV is an important protein which is essential for the development of a healthy immune system, as well as for the protection of genomic integrity. It is encoded in the LIG4 gene. Complete knock-out of the LIG4 gene in mice resulted in embryonic lethality [12, 13]. This finding suggests that a null mutation might also be nonviable in human beings and explains why, until now, only hypomorphic mutations of the LIG4 gene have been described [11, 14]. Depending on the expression of the hypomorphic mutation, the immunologic phenotype ranges from T-B-NK+ SCID (T cell-negative (T-), B cell-negative (B-), natural killer cell-positive (NK+)) to milder degrees of lymphopenia and hypogammaglobulinemia [5, 10, 15]. There are several other conditions with overlapping features such as Nijmegen breakage syndrome, Cernunnos-XLF deficiency, Fanconi anemia and Seckel syndrome [11].

The initial treatment aims to prevent life-threatening infections by administering antibiotic, antiviral and antifungal chemoprophylaxis, as well as transfusing immunoglobulins and blood (components) on a regular basis. Non-essential ionizing radiation should be avoided [11]. However, in severe cases with progressive bone marrow failure and the T-B-NK+ SCID phenotype this might not be sufficient. The risk of malignancy rises over time. HSCT could be a curative treatment option to prevent serious infections and deadly hematopoietic malignancies [10, 11].

In accordance with relevant current literature [6, 10, 15–20], this report illustrates that HSCT in patients with DNA LIG4 deficiency can result in very different outcomes. The unique addition to these descriptions is that two brothers with the same underlying mutation received two different treatment protocols. While a rather mild conditioning regimen was successful in one brother, a rather more aggressive regimen failed in the other. Therefore, further studies are required to examine the role of different conditioning protocols and immunosuppressive treatments as well as the efficacy of this treatment option in ligase IV deficiency.

## Case presentation

### Family medical history

Three children were born to Caucasian, non-consanguineous parents, who were in good health. There was no history of hereditary disorders in the family. The oldest child, a girl, was healthy and developed according to age.

### Case 1

In June 2007, at the age of 2 years and 7 months, the older brother was referred to the hemato-oncological department of the University Children’s Hospital Tuebingen. He presented with microcephaly and remarkable growth retardation (weight 10.3 kg (<3rd percentile), length 83 cm (<3rd percentile), head circumference 44 cm (<3rd percentile)). His skin appeared scaly, dry and rather pale; his hair was dry, brittle and scarce. Furthermore, the patient suffered from chronic bloody diarrhea, due to a persistent infection with norovirus. No neurological deficits were detected. The laboratory findings revealed leucopenia (white blood cells 2900/μl), anemia (hemoglobin 6.8 g/dl) and mild thrombocytopenia (thrombocytes 168,000/μl). The number of lymphocytes (484/μl) was reduced, with CD3+ T cells on the lower limit (282/μl), no B cells (CD19+ 1/μl), sufficient CD4+ T cells (214/μl), remarkably few CD8+ T cells (34/μl) and reduced CD16/56+ NK cells (81/μl). The immunoglobulin levels were within the normal range (IgG 924 mg/dl, IgA 112 mg/dl, IgM 63 mg/dl). The subclass analysis of IgG showed very low levels of IgG2 (IgG1 705 mg/dl, IgG2 43 mg/dl, IgG3 24 mg/dl, IgG4 < 7 mg/dl). Conspicuously, the boy did not show adequate antibody titers after having received the standard vaccinations for his age. The first year of the boy’s life had been unremarkable. But during his second year of life the patient suffered many viral and bacterial infections, mainly affecting the respiratory and the urinary tract. Cytomegalovirus (CMV) was detected several times in the urine and norovirus in the stool. There were several episodes of unclear fever. The boy had to be hospitalized multiple times due to severe infections and was treated with antibiotics eight times in 1 year. Furthermore the failure to thrive became obvious as well as a mild hepatomegaly. Several differential diagnoses such as cystic fibrosis, coeliac disease, gastrointestinal diseases or severe underlying infections such as hepatitis were ruled out in the previously treating hospital. As an immunodeficiency syndrome was suspected, he was treated with antibiotic chemoprophylaxis and intravenous immunoglobulins, starting when he first presented to the Children’s Hospital Tuebingen. In order to identify the type of immunodeficiency syndrome a genetic analysis was performed in June 2007. At the age of 32 months the genetic findings revealed a DNA ligase IV deficiency, associated with SCID, with a compound heterozygous mutation in exon two of DNA LIG4 gene ((NM_002312) exon 2: c.613delT p.S205LfsX29 heterozygous and exon 2: c.845A > T p.H282L heterozygous). Both parents carry a heterozygous mutation (mother: ligase IV exon 2: c.845A > T p.H282L, heterozygous; father: ligase IV exon 2: c.613delT p.S205LfsX29 heterozygous). The sequencing of the RAG-1/2, Artemis, XLF and TCF3 genes did not show any abnormalities. In the chromosomal analysis a balanced translocation (46, XY, t (1;19) (q21; p13)) was found. The same karyotype could be detected in the healthy father. Thus, this chromosomal defect is considered an incidental finding.

**Fig. 1:**
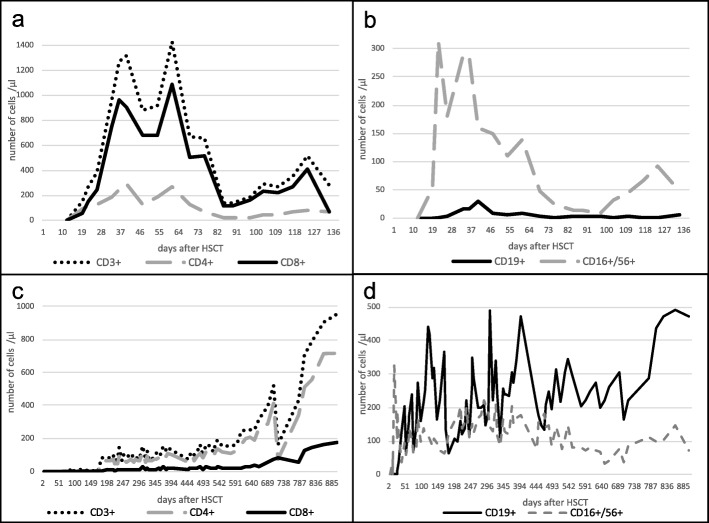
Lymphocyte subsets by flow cytometry for T cells, B cells and NK cells after HSCT in both cases. **a** Development of T cells (CD3+, CD4+, CD8+) after HSCT in case 1, the number of T cells is decreasing over time. **b** Development of B cells (CD19), and NK cells (CD16+/56+) after HSCT in case 1, the number of B cells and NK cells is decreasing over time. **c** Development of T cells (CD3+, CD4+, CD8+) after HSCT in case 2. In contrast to case 1, the number of T cells is rising over time in case 2. **d** Development of B cells (CD19), and NK cells (CD16+/56+) after HSCT in case 2. In contrast to case 1, the number of B cells and NK cells is rising over time in case 2. Standard values: CD3+ (800–1000/μl), CD4+ (~ 400/μl), CD8+ (~ 400/μl), CD19+ (200–400/μl), CD16+/56+ (~ 200/μl)

In August 2007, at the age of 33 months, HSCT was performed due to the severity of the immunodeficiency. Conditioning was myeloablative with fludarabine (4 × 40 mg/m^2^), thiotepa (1 × 10 mg/kg BW), melphalan (2 × 70 mg/m^2^) and anti-thymocyte globulin (10 mg/kg BW, divided 1 × 1 mg/kg BW, 3 × 3 mg/kg BW). The boy suffered from severe mucositis during aplasia. He received 8.77 × 10^6^/kg BW CD34+ hematopoietic progenitor cells, 1.524 × 10^7^/kg BW CD3+ cells derived from the bone marrow of an HLA-identical (10/10 match), non-related female donor. The graft-versus-host disease (GvHD) prophylaxis was carried out with CSA (cyclosporine A 3 mg/kg BW, target level 150–200 ng/ml, day − 1 until death on day + 142), and methotrexate 10 mg/m^2^ (day + 1, + 3, + 6). Leukocyte and granulocyte take was observed at day + 15. In the following days, the child developed acute GvHD of the skin (II°-III°, day + 17) which was initially treated locally with tacrolimus and glucocorticoids and then systemically with prednisolone (5 mg/kg BW). After an initial improvement, the GvHD of the skin relapsed and was then treated with methylprednisolone (5 mg/kg BW). A slow recovery was observed. Further complications, due to HSCT, were a foscavir-associated ulcer of the penis, CSA-associated hypertension, a reactivation of Epstein-Barr virus (EBV) and CMV, as well as a possible candidemia (positive serology for *Candida* antigen in the peripheral blood three times) without any clinical symptoms. Treatment was changed from amphotericin B to caspofungin. Complete donor chimerism was observed 1 month after HSCT. On day + 32, 5690/μl WBC, 1414/μl lymphocytes, (960/μl CD3+, 16/μl CD19+, 189/μl CD4+, 752/μl CD8+, 291/μl CD16/56+) were detected in the peripheral blood (Fig. 1).

**Fig. 2:**
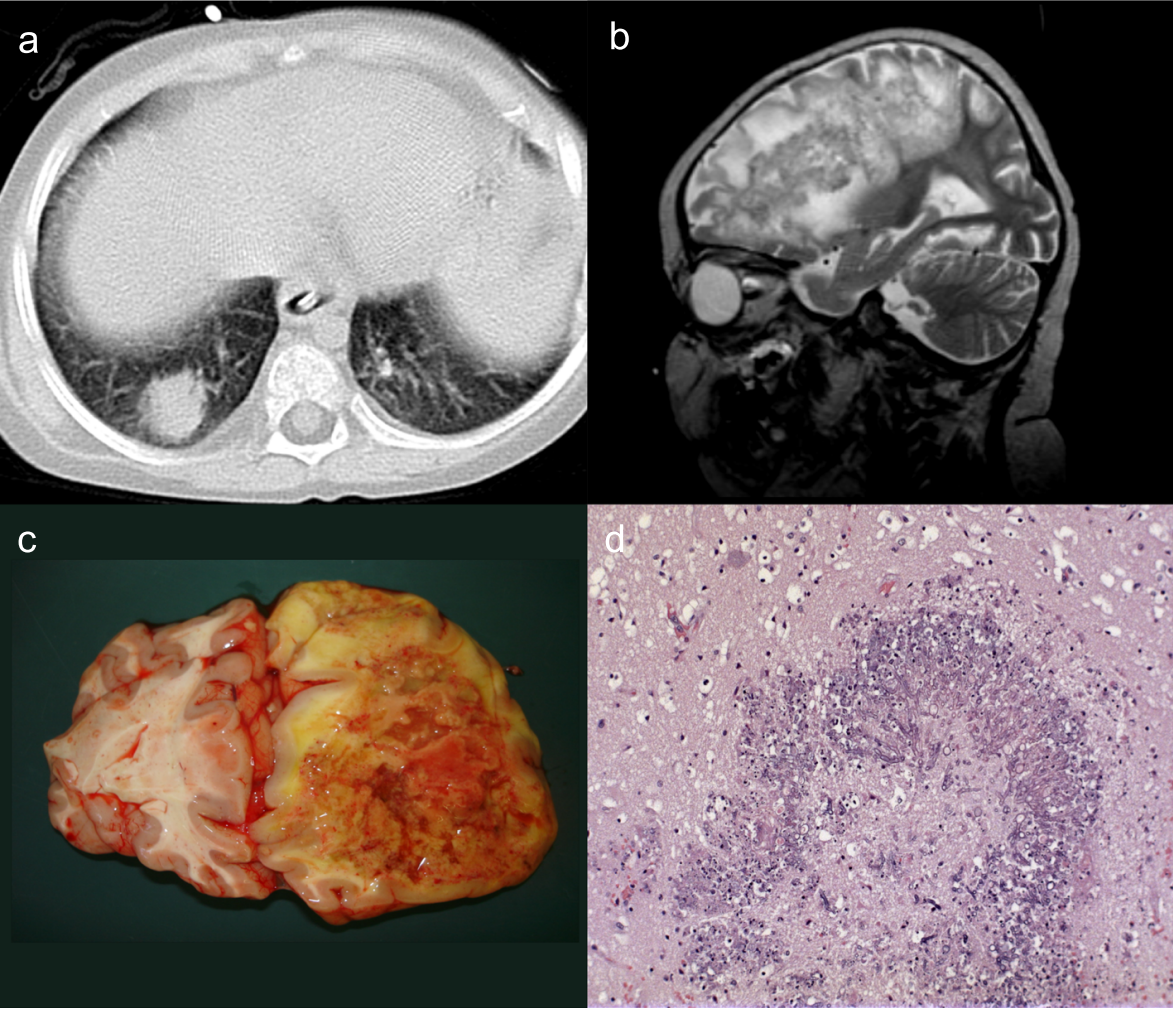
Multifocal aspergillosis in case 1. **a** HRCT of the lung base demonstrates a large pulmonary nodule in the right lower lobe surrounded by a small halo. **b** Sagittal T2w MRI shows an overlap pattern of cerebritis and abscess within the right hemisphere. Note the multiple small hypo-intense foci which represent hemorrhages. **c** Neuropathological findings: macroscopic picture of the brain with a necrotizing brain abscess in the right frontal lobe, caused by aspergillosis. **d** Neuropathological findings: H&E stain: histological brain tissue with central necrosis surrounded by y-shaped hyphae, consistent with aspergillosis

A veno-occlusive disease (VOD) of the liver was diagnosed on day + 58 and on day + 74 the boy developed severe acute intestinal GvHD stadium IV, with bloody and watery diarrhea (stool volume > 1000 ml/m^2^ body surface area per day). The symptoms stabilized for a short time with high-dose methylprednisolone (10 mg/kg BW per day, for 3 days), but then relapsed again. Further treatment consisted of somatostatin-infusions and symptomatic substitution of thrombocytes, erythrocytes, fresh frozen plasma (FFP) and coagulation factors. A causal immunosuppressive combination therapy, consisting of tacrolimus, steroids, CSA and infliximab was unable to stop the intestinal bleeding sufficiently. Furthermore the boy received 3.7 × 10^6^/kg BW mesenchymal stem cells from his father as GvHD treatment. A colonoscopy showed necrosis and ulcers of the colonic mucosa with diffuse bleeding. A probable pulmonary aspergillosis was detected on day + 105. *Aspergillus*-galactomannan-antigen was positive (1.9 EIA, rising quickly to 4.8 EIA) and the computed tomography (CT) scan showed intrapulmonary noduli suspected to be aspergillomas (Fig. 2a). Therefore, amphotericin B was added to the antifungal treatment with caspofungin. On day + 135, a severe intestinal bleeding, associated with intestinal GvHD, occurred (hemoglobin decreased from 14 g/dl to 5 g/dl) and the patient was transferred to the intensive care unit (ICU) for massive transfusions of blood, thrombocytes, FFP and coagulation factors. He suffered from acute renal failure and transfusion-related acute lung injury. In combination with the aspergillosis, this led to an acute pulmonary hemorrhage and the need for mechanical ventilation.

Moreover, while being transferred to the ICU, the boy was already suffering from hemiparesis and somnolence. These neurological symptoms worsened and a magnetic resonance imaging (MRI) was conducted. The scan showed severe brain damage that could be associated with a cerebral aspergillosis (Fig. 2b) although *Aspergillus* antigen was not detected in the cerebrospinal fluid. On day + 143 after HSCT, the patient succumbed to multiple organ failure. The autopsy report confirmed the suspected, invasive, cerebral aspergillosis (Fig. 2c, d).

### Case 2

Another boy was born into the family in November 2006. Although this boy, 2 years younger than his brother, was hypotrophic at birth (weight 2.610 kg (<3rd percentile), length 49 cm (7th percentile), head circumference 33 cm (<3rd percentile)), the first 3 months of his life were uneventful. Subsequently, numerous pulmonary infections and chronic bronchitis with cough, pulmonary obstruction and secretion occurred. As with his brother, the laboratory findings revealed leucopenia (WBC 2400/μl) anemia (hemoglobin 7.9 g/dl) and mild thrombocytopenia (207,000/μl). The number of lymphocytes was reduced (245/μl) with extremely reduced counts of B, T, and NK cells (CD3+ 70/μl, CD19+ 2/μl, CD4+ 51/μl, CD8+ 13/μl, CD16/56+ 11/μl). With the exception of IgA (< 6 mg/dl), the immunoglobulin levels were within the normal range (IgG 395 mg/dl, IgM 46 mg/dl). The subclass analysis of IgG was normal (IgG1 279 mg/dl, IgG2 63 mg/dl, IgG3 30 mg/dl, IgG4 < 7 mg/dl). Similar to his older brother, the boy was treated with antibiotic, antifungal, and antiviral chemoprophylaxis and intravenous immunoglobulins. At the age of 8 months, the diagnosis of DNA ligase IV deficiency was confirmed genetically, at the same time as his brother was diagnosed. He carries the same compound heterozygous mutation in exon two of LIG4 gene (exon 2: c.613delT p.S205LfsX29 heterozygous and exon 2: c.845A > T p.H282L heterozygous) and the same karyotype (46, XY, t (1; 19) (q21; p13)).

**Fig. 3:**
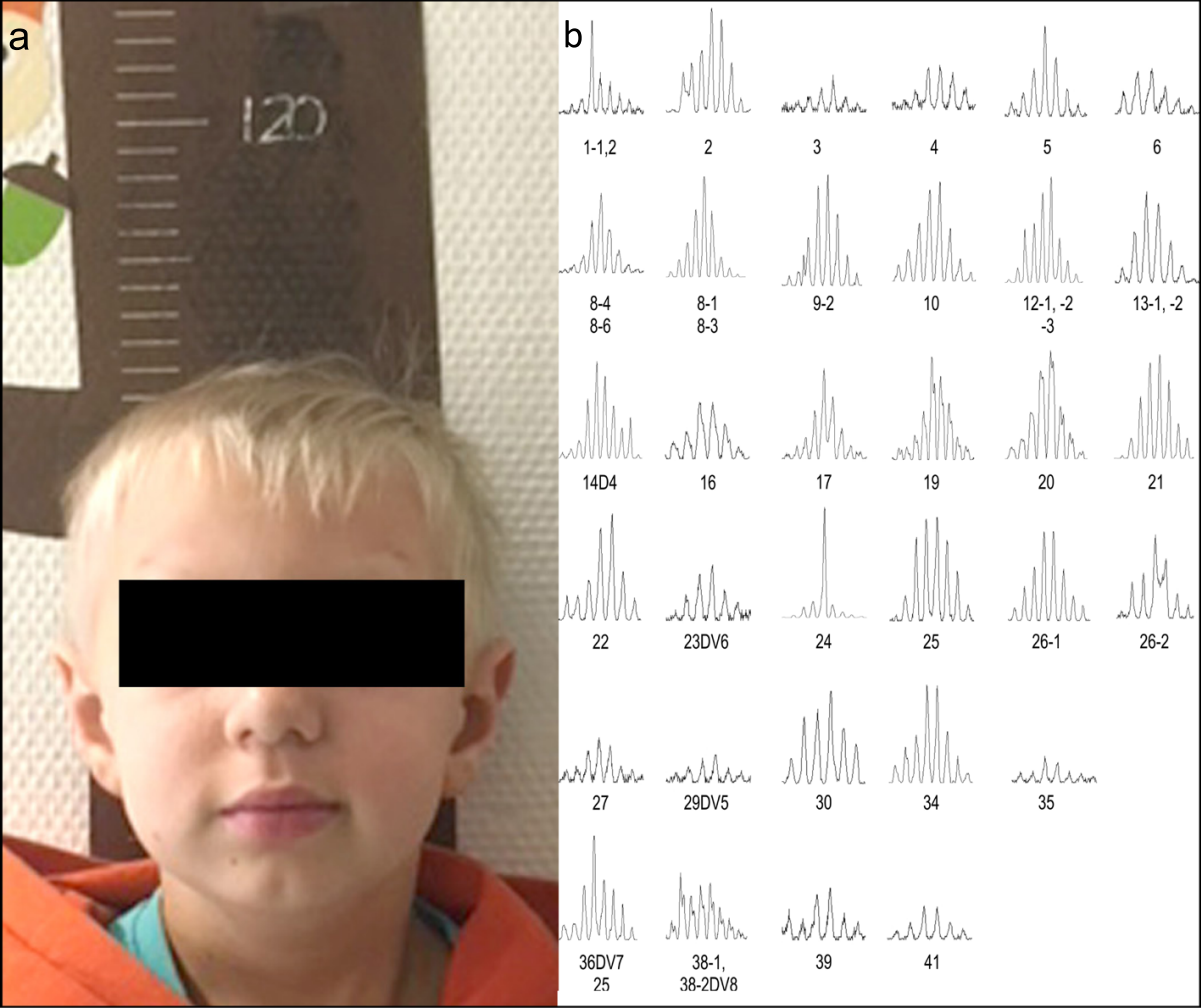
Follow-up 10 years after HSCT. **a** Picture of the boy, 10 years after HSCT: bird-like face with sloping forehead, micrognathia, long nose, microcephaly (head circumference 45.1 cm (< 3rd percentile)) and dwarfism (length 110.8 cm (27 cm (< 3rd percentile)). **b** Spectratype analysis of TCR Vα repertoire of peripheral T cells. Assessment of T cell receptor diversity for monitoring immune reconstitution post-hematopoietic stem cell transplantation in case 2. The spectratype analysis shows a complete, unbiased and broad based repertoire indicating a maximum of TCR diversity, conferring immune competence

At the age of 18 months, chronic, infiltrative changes in the lung structure were identified in the CT scan. Furthermore, there was a progressive bone marrow failure. Based on those findings, HSCT was performed. The treatment protocol was adapted because his brother had suffered from severe GvHD. This time, allogeneic HSCT was performed with a combination of dosage-reduced, myeloablative conditioning and T cell-depletion (CD3/CD19 depletion) as well as CD34-selection. The chemotherapy consisted of fludarabine (4 × 40 mg/m^2^), melphalan (2 × 60 mg/m^2^) and anti-thymocyte globulin (10 mg/kg BW, divided 1 × 1 mg/kg BW, 3 × 3 mg/kg BW). Apart from a skin reaction with depigmentation, chemotherapy was tolerated well. There was no mucositis. The patient received 40 × 10^6^/kg BW CD34+ hematopoietic progenitor cells and 1.62 × 10^4^/kg BW CD3+ cells from a HLA-identical (10/10 matched), unrelated male donor. Besides the T cell-depletion no other GvHD prophylaxis was performed. Leukocyte and granulocyte take was observed at day + 10. On day + 29, complete chimerism and 1960/μl white blood cells (0/μl CD3+, 11/μl CD19+, 0/μl CD4+, 0/μl CD8+, 189/μl CD16/56+) were observed in the peripheral blood (Fig. 1c, d). There were no severe infections or GvHD post-transplant. About 6 months after the HSCT, the patient suffered from hemolytic uremic syndrome with acute renal failure, hypertension and edemas. The child developed a dilated cardiomyopathy due to hypertension. These symptoms were treated and disappeared during the following years. The cell count for B and T cells has normalized during the past couple of years (Fig. 1c, d), and the current T cell receptor repertoire, analyzed via Vα spectratype, shows a polyclonal diversity of T cell receptors reflecting a healthy immune competence (Fig. 3b). Today, the boy is 12 years old and is living a normal life. He attends a regular high school, plays sports and enjoys a good quality of life. At the 10-year follow up, he presented with extreme dwarfism and dystrophy (weight 14.1 kg (14.5 kg <3rd percentile), length 110.8 cm (27 cm <3rd percentile) head circumference 45.1 cm (<3rd percentile)) (Fig. 3a). The bone age, calculated according to Greulich and Pyle [21], was in accordance with the chronological age. His maximum final adult height was calculated to be 135 cm. The laboratory findings showed normal blood counts with hemoglobin 13.4 g/dl, thrombocytes 277,000/μl and WBC 9900/μl with 4059/μl lymphocytes (2030/μl CD3+, 755/μl CD19+, 1023/μl CD4+, 398/μl CD8+, 321/μl CD16/56+). Without any substitution the immunoglobulin levels were generally low, but for IgA and IgM within the normal range; only IgG remained below the standard value (IgG 411 mg/dl, IgA 87 mg/dl, IgM 63 mg/dl) at the 10-year follow up. Complete donor chimerism was observed. The endocrinological findings revealed a hypergonadotropic hypogonadism (luteinizing hormone 6.4 IU/l (normal range: 1–3.5 IU/l), follicle-stimulating hormone 64.4 IU/l (normal range: < 0.2–7.5 IU/l)). The echocardiographic examination showed further improvement with very mild residuals of the dilated cardiomyopathy.

## Discussion and conclusions

Generally there is a broad spectrum of clinical manifestation associated with LIG4 deficiency. The compound heterozygous mutations (c.613delT, c.845A > T) in both brothers are not among the most common in ligase IV deficiency but have been mentioned before individually [3, 7, 16, 17]. However, no combination of the two variants in one patient has been mentioned in the literature before. Considering the classification of *the American College of Medical Genetics and Genomics an the Association for Molecular Pathology* [22] the mutation c.613delT has to be classified as ‘pathogenic’. It results in a frameshift and a premature stop codon in the DNA-binding domain (p.S205LfsX29). This mutation is described in the literature in two patients with ligase IV deficiency and in a patient presenting with Dubowitz syndrome [3, 23, 24]. The missense variant c.845A > T inherited by the mother is graded as ‘likely pathogenic’. The reference transcript p.H282L has been described in patients receiving HSCT (Table 1).

**Table 1:** Summary of the literature. Conditioning regimen, GvHD prophylaxes, mutations and outcome of the published case reports of HSCT in patients with ligase IV deficiency

patient	conditioning regimen	GvHD prophylaxes	outcome	mutation	ref.
girl, 2 months	N/A	N/A	died 2 months after HSCT due to VOD	c.1544_1548del5bp (p.K424fs20X), c.1112A > G (p.Q280R)	[18]
girl, 19 months	N/A	N/A	died due to EBV-associated lymphoproliferative syndrome on day + 50	c.1544_1548del5bp (p.K424fs20X), c.1112A > G (p.Q280R)	[18]
girl, 11 years	fludarabine (4 × 30 mg/m^2^), cyclophosphamide (4 × 10 mg/kg), anti-thymocyte globulin (4 × 15 mg/kg)	cyclosporine A	survived	c.1406G > A (p.G469E), c.2440C > T (p.R814X)	[10]
girl, 10 years	fludarabine (5 × 35 mg/m^2^), cyclophosphamide (4 × 10 mg/kg), anti-thymocyte globulin	cyclosporine A	survived, autologous reconstitution, day + 23	c.1762delAAG (p.K588del), homozygous	[19]
same girl, 10 years	busulfan (4 × 2 mg/kg), cyclophosphamide (3 × 50 mg/kg), anti-thymocyte globulin	methotrexate, cyclosporine A	survived	c.1762delAAG (p.K588del), homozygous	[19]
girl, 4 months	fludarabine, thiotepa	cyclosporine A	survived	c.1118A > T (p.H282L), c.1544_1548delAAAGA (p.D423fs442X)	[16]
girl, 2 years	N/A	N/A	died during preparative therapy for HSCT	c.1118A > T (p.H282L), c.1544_1548delAAAGA (p.D423fs442X)	[16]
girl, 6 months	busulfan (4 × 16 mg/kg), cyclophosphamide, (4 × 200 mg/kg)	cyclosporine A, methylprednisolone, mycophenolat mofetil	survived	c.845A > T (p.H282L), c.1747_1751del5bp (p.R581fsX)	[17]
girl, ~ 2 years	busulfan (4 × 50 mg), cyclophosphamide, (4 × 500 mg)	N/A	died due to bradycardia and respiratory arrest	g.5333_5335delCAA (p.Q433del), homozygous	[15]
boy, 4 years	alemtuzumab (3 × 0,3 mg/kg), anti CD45 (4 × 0,4 mg/kg), fludarabine (5 × 30 mg/kg), cyclophosphamide (4 × 300 mg/m^2^)	cyclosporine A, mycophenolat mofetil	survived	N/A	[20]
girl, 19 months	cyclophosphamide (10 mg/kg), fludarabine (30 mg/m^2^), anti-thymocyte globulin (15 mg/kg)	cyclosporine A, prednisolone	died due to toxic epidermal necrolysis and fungal infection	c.1904delA, c.907G > C	[6]
boy, 33 months	fludarabine (4 × 40 mg/m^2^), thiotepa (1 × 10 mg/kg), melphalan (2 × 70 mg/m^2^), anti-thymocyte globulin (10 mg/kg)	cyclosporine A, prednisolone, methotrexate	died due to aspergillosis	c.845A > T (p.H282L), c.613delT (p.S205LfsX29)	this case
boy, 18 months	fludarabine (4 × 40 mg/m^2^), melphalan (2 × 60 mg/m^2^), anti-thymocyte globulin (10 mg/kg)	T cell-depletion (CD3/CD19 depletion)	survived	c.845A > T (p.H282L), c.613delT (p.S205LfsX29)	this case

Abbreviations: *N/A* Not applicable

There is no established genotype-phenotype correlation. The disease severity is assumed to be associated with the residual activity of ligase IV, but influenced by the DNA repair mechanisms [8]. In general biallelic truncating mutations are associated with a more severe phenotype than compound-heterozygous localization of a missense and a truncating variant [3]. The combination of different variants can be classified in genotype 1 (two truncating mutations) and genotype 2 (truncating mutation and missense mutation) with different immunoglobulin levels. Genotype 2 is associated with a mild to moderate ligase IV deficiency [7]. Patients carrying missense mutations show remarkably reduced function of the ligase IV [11]. p.H282L leads to a residual ligase IV enzyme activity of 5 to 10% [25]. The paternal mutation (c.613delT p.S205LfsX29) is associated with a severe form of ligase IV deficiency, characterized by early mortality and SCID. This can be explained by the correlation between the location of truncating LIG4 variants and the severity of the resulting phenotype in patients with two truncating variants studied by Murray et al.. A late truncating mutation is associated with rather mild symptoms, whereas early truncating mutations such as p.S205LfsX29 are associated with a severe growth retardation and immunodeficiency [3]. In one report, the authors even concluded that this mutation might result in a null mutation because of the lack of a nuclear localization signal [23]. These observations are consistent with the presented case of a severe type of ligase IV deficiency with major infections and high mortality. In contrast, an intrafamilial variability of the phenotype was demonstrated in two unrelated families in siblings carrying genotype 2 [5, 16]. In addition two patients from another study with the same genotype 2 displayed slightly variable phenotypes and one of them developed non-Hodgkin lymphoma [2]. Thus there might be additional factors influencing the manifestation and severity in the phenotype of ligase IV deficiency. For instance there is a report on a somatic reversion of germline LIG4 variant in some clones of blood cell lines detected in one patient with LIG4 deficiency [2].

To date, no curative treatment option exists. However, HSCT has been proposed as a therapy option [11]. By transplanting a new immune system, the risk of life-threatening infections and malignancies can be reduced, as well as the need for transfusions. Other symptoms such as microcephaly, growth retardation, “bird-like” face or developmental and mental delay are not affected by HSCT [17].

In the presented case, only one of the two brothers survived HSCT. High mortality in this particular group of patients is consistent with current literature. There are 11 published case reports of HSCT in DNA ligase IV deficiency [6, 9–11, 15–20, 26]. Out of these cases, six survived HSCT and five died due to severe side-effects of the conditioning regimen (Table 1). The older brother’s diagnosis was unknown for a long time, resulting in late referral into a specialized unit. Consequently HSCT was performed rather late (33 months), compared to the published cases (median 24 months, range 2–132 months). Based on the limited number of reported cases involving various mutations no correlation between position of mutation and outcome after HSCT can be derived.

As there was no HLA-identical family donor, the two brothers received HSCT from a matched unrelated donor (MUD). A retrospective analysis of HSCT in 94 SCID-patients concluded that if there was no HLA-identical family donor, a MUD would be the best option. There might be a better engraftment, reconstitution, quality of life and overall survival, when using a graft from a MUD compared to a mismatched related donor [17, 27, 28].

The conditioning regimen is especially important in patients with DNA ligase IV deficiency, because there is increased toxicity in patients suffering from DNA repair disorders. Thus, chemotherapy-based conditioning is poorly tolerated and may result in transplant-related morbidity and mortality [20]. To date, there is no recognized standard conditioning regimen of HSCT in patients with DNA ligase IV deficiency. A review of published case reports, indicates that several chemotherapy regimens have been administered, either myeloablative or in reduced intensity [6, 10, 15–17, 19, 20, 29]. A summary of the published literature is displayed in Table 1.

The regimen of the older brother has been used in another case as well. This patient survived HSCT, but suffered from severe hemolytic-uremic syndrome [16]. Furthermore, a reduced-intensity conditioning regimen consisting of fludarabine (4 × 30 mg/m^2^), cyclophosphamide (4 × 10 mg/kg BW) and anti-thymocyte globulin (4 × 15 mg/kg BW) is described. One patient went through this mild regimen without major problems [10]. Another patient died 1 month post-transplant, due to toxic epidermal necrolysis and fungal respiratory infection [6]. If the conditioning regimen is too mild, the risk of autologous reconstitution will increase. This effect was observed in one patient who received the same, intensity-reduced conditioning regimen. The patient subsequently underwent a second HSCT with chemotherapy consisting of busulfan, cyclophosphamide and anti-thymocyte globulin. This time, the transplantation was successful [19]. The myeloablative combination of busulfan and cyclophsphamid has been applied in another HSCT successfully [17]. In contrast, an 18-months-old girl died due to bradycardia and respiratory arrest during the conditioning process, consisting of the same regimen [15]. Generally, there are different approaches to modify the conditioning regimen. An antibody-based minimal-intensity conditioning regimen consisting of anti-CD45 monoclonal antibodies, alemtuzumab (anti-CD52), fludarabine and low-dose cyclophosphamide is described. Out of 16 high-risk patients treated by this combination 81% survived and were cured [20].

### Review of the published case reports of HSCT in ligase IV deficiency

Due to the rarity of DNA ligase IV deficiency and the limited number of patients undergoing HSCT, there is no general recommendation as to which conditioning protocol should be followed. Because of the severe radiosensitivity, irradiation should not be part of the conditioning regimen [11, 19]. Patients with defects in NHEJ suffer from an increased sensitivity to alkylating agents. For best survival and minimal late effects, it is suggested to minimize the exposure to alkylating agents. In accordance with our report, myeloablative conditioning does not seem to be necessary for performing HSCT successfully. On the contrary, myeloablative conditioning regimens are associated with a higher mortality and morbidity compared to reduced intensity conditioning regimens [29, 30]. In the future, transplantation of gene-corrected autologous CD34+ cells might simplify the conditioning regimen [31].

Another major difference between the two cases is the GvHD prophylaxis. In the first case, the prophylaxis was carried out with CSA (cyclosporine A, day − 1 until death) and methotrexate 10 mg/m^2^ (day + 1, + 3, + 6). In contrast, there was no GvHD prophylaxis apart from CD3/CD19 depletion and anti-thymocyte globulin in the second case. In an experimental in vitro study, it was shown that CSA can induce double-strand breaks in cells with DNA ligase IV deficiency. Furthermore, double-strand breaks particularly occurred in busulfan and fludarabine pre-treated DNA LIG4 deficient cells [32]. Fludarabine was part of the chemotherapy in the first case. Moreover the dosage of CSA (3 mg/kg) was rather low in successfully transplanted cases [10, 19]. The role of CSA in HSCT in patients with DNA LIG4 deficiency has still to be determined in further studies, but it might have a negative impact on the outcome in this selected group of patients inducing double-strand breaks.

In the 10-year follow up, the younger brother was in good shape, except for the ligase IV deficiency-related dwarfism and dystrophy. A fully recovered immune system, was confirmed by Vα spectratype (Fig. 3b). Hypergonadotropic hypogonadism was an unexpected finding. To the authors’ knowledge, there is no proven association between ligase VI deficiency and hypergonadotropic hypogonadism. But there are four descriptions of hypogonadism in LIG4 deficiency patients [3, 9, 11, 19]. Further investigation is needed to clarify if there are some endocrinological disorders associated with LIG4 deficiency. Thus, the cause of this endocrinological problem in the boy remains unknown. The laboratory findings indicate a primary testicular damage. One might speculate that it could also be related to HSCT’s toxicity, or to the late orchidopexy, which was performed at the age of 4 years.

The data presented in this case report does not allow any generalization concerning treatment recommendations or cause-effect relationships as this is a retrospective description of two patients with a rare genetic disease. The strength of this case can be found in the fact that two brothers sharing the same genetic background and who are suffering from an identical mutation are treated with different HSCT regimen resulting in vastly different outcomes with one brother dying and the other one - receiving a milder regimen - fully recovering. Therefore further studies to carefully evaluate the efficacy of HSCT as a treatment option, as well as the conditioning regimen and GvHD prophylaxes in patients with DNA ligase IV deficiency are required.

## Data Availability

The datasets used and/or analyzed in the current study are available from the corresponding author on reasonable request.

## Abbreviations

BW: Bodyweight
CMV: Cytomegalovirus
CSA: Cyclosporine A
CT: Computed tomography
DNA: Deoxyribonucleic acid
EBV: Epstein-Barr virus
FFP: Fresh frozen plasma
GvHD: Graft-versus-host disease
HSCT: Hematopoietic stem cell transplantation
ICU: Intensive care unit
LIG4: DNA ligase IV
MRI: Magnetic resonance imaging
MUD: Matched unrelated donor
NHEJ: Nonhomologous end-joining
SCID: Severe combined immunodeficiency
TCR: T cell receptor
VOD: Veno-occlusive disease
WBC: White blood cells
XLF: XRCC4-like factor
