# Correction to: Allogeneic hematopoietic stem cell transplantation in two brothers with DNA ligase IV deficiency: a case report and review of the literature

**DOI:** 10.1186/s12887-019-1851-6

**Published:** 2019-12-02

**Authors:** Sarah Schober, Karin Schilbach, Michaela Doering, Karin M. Cabanillas Stanchi, Ursula Holzer, Patrick Kasteleiner, Jens Schittenhelm, Juergen F. Schaefer, Ingo Mueller, Peter Lang, Rupert Handgretinger

**Affiliations:** 1 Department I – General Pediatrics, Hematology/Oncology, University Children’s Hospital Tuebingen, Hoppe-Seyler-Str.1, 72076 Tuebingen, Germany; 2 Department of Neuropathology, Institute of Pathology and Neuropathology, Eberhard-Karls University Tuebingen, Calwer Str. 3, 72076 Tuebingen, Germany; 3 Department of Diagnostic and Interventional Radiology, University Hospital Tuebingen, Hoppe-Seyler-Str. 3, 72076 Tuebingen, Germany; 4 Division for Pediatric Stem Cell Transplantation and Immunology, Clinic for Pediatric Hematology and Oncology, University Medical Center Hamburg-Eppendorf, Martinistr. 52, 20246 Hamburg, Germany

**Correction to: BMC Pediatr (2019) 19:346**

**https://doi.org/10.1186/s12887-019-1724-z**

After publication of the original article [1], it was brought to our attention that references 24 and 31 are cited incorrectly in the article.

Reference ‘[2]’ (see reference list below) should be cited where reference 24 is cited; NB the correct reference [2] is missing in the original article.

While reference ‘[3]’ (see reference list below) should be cited where reference 31 is cited; NB the correct reference [3] is present in the original article, but in the original article it is reference ‘29’.

The authors apologize for any inconvenience caused.
